# Supplementary material for: Splice-Junction-Based Mapping of Alternative Isoforms in the Human Proteome
Source: Cell Rep. Author manuscript; Available in PMC 2020 Jan 15. (PMC6961840; doi:10.1016/j.celrep.2019.11.026)
Supplement: 2 [file NIHMS1546469-supplement-2.zip › DF1/PXD009737/TestisGluC/TestisGluC_23_ARAP1_RVMTKKMTLTTMRSQRRGRGPQPESVAEWLRALHLE.pdf]

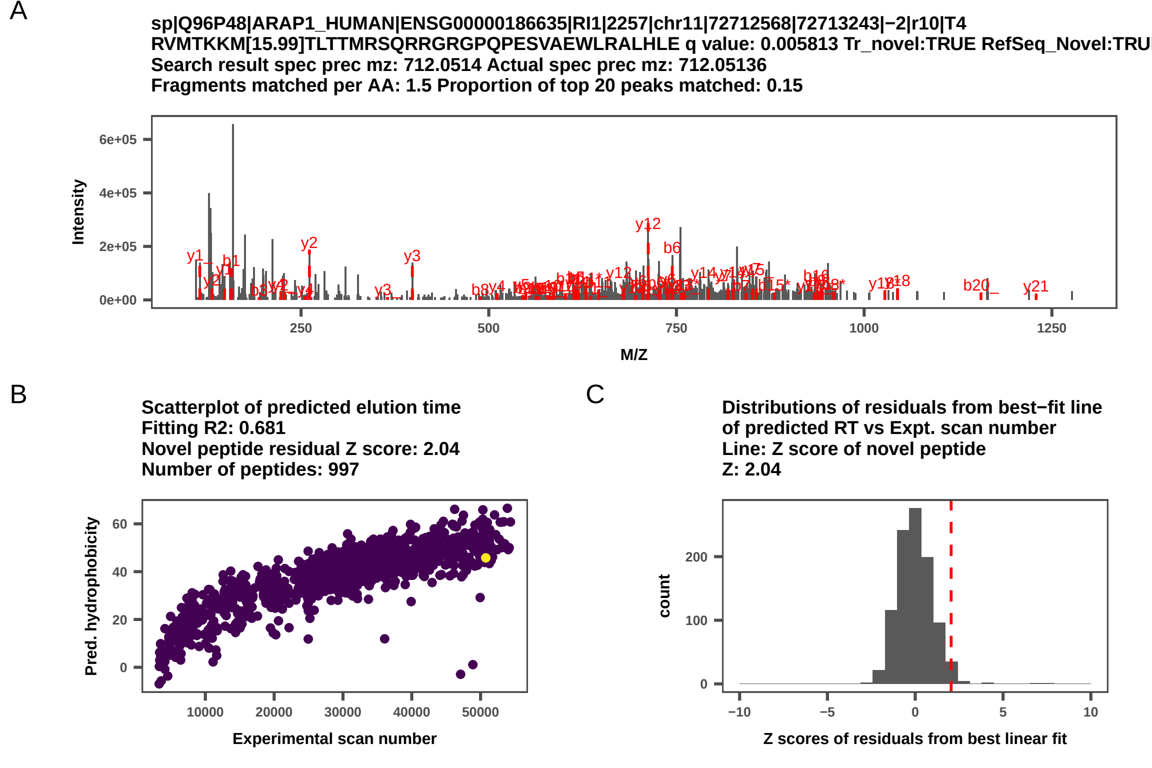
A
sp|Q96P48|ARAP1_HUMAN|ENSG00000186635|RI1|2257|chr11|72712568|72713243|−2|r10|T4
RVMTKKM[15.99]TLTTMRSQRRGRGPQPESVAEWLRALHLE q value: 0.005813 Tr_novel:TRUE RefSeq_Novel:TRU
Search result spec prec mz: 712.0514 Actual spec prec mz: 712.05136
Fragments matched per AA: 1.5 Proportion of top 20 peaks matched: 0.15
Intensity
6e+05
4e+05
2e+05
0e+00
y1
b1
y2
y3
y12
b6
b20
y21
250
500
750
1000
1250
M/Z
B
Scatterplot of predicted elution time
Fitting R2: 0.681
Novel peptide residual Z score: 2.04
Number of peptides: 997
Pred. hydrophobicity
60
40
20
0
10000
20000
30000
40000
50000
Experimental scan number
C
Distributions of residuals from best−fit line
of predicted RT vs Expt. scan number
Line: Z score of novel peptide
Z: 2.04
count
200
100
0
−10
−5
0
5
10
Z scores of residuals from best linear fit
